# Parkinson’s Disease Detection from Voice Recordings Using Associative Memories

**DOI:** 10.3390/healthcare11111601

**Published:** 2023-05-30

**Authors:** Irving Luna-Ortiz, Mario Aldape-Pérez, Abril Valeria Uriarte-Arcia, Alejandro Rodríguez-Molina, Antonio Alarcón-Paredes, Elías Ventura-Molina

**Affiliations:** 1 Instituto Politécnico Nacional, Center for Computing Innovation and Technological Development (CIDETEC), Computational Intelligence Laboratory (CIL), Mexico City 07700, Mexico; 2 Tecnológico Nacional de México/IT de Tlalnepantla, Research and Postgraduate Division, Tlalnepantla de Baz 54070, Mexico; 3 Instituto Politécnico Nacional, Center for Computing Research (CIC), Computational Intelligence Laboratory (CIL), Mexico City 07700, Mexico

**Keywords:** associative memories, decision support systems, Parkinson’s disease classification

## Abstract

Parkinson’s disease (PD) is a neurological condition that is chronic and worsens over time, which presents a challenging diagnosis. An accurate diagnosis is required to recognize PD patients from healthy individuals. Diagnosing PD at early stages can reduce the severity of this disorder and improve the patient’s living conditions. Algorithms based on associative memory (AM) have been applied in PD diagnosis using voice samples of patients with this health condition. Even though AM models have achieved competitive results in PD classification, they do not have any embedded component in the AM model that can identify and remove irrelevant features, which would consequently improve the classification performance. In this paper, we present an improvement to the smallest normalized difference associative memory (SNDAM) algorithm by means of a learning reinforcement phase that improves classification performance of SNDAM when it is applied to PD diagnosis. For the experimental phase, two datasets that have been widely applied for PD diagnosis were used. Both datasets were gathered from voice samples from healthy people and from patients who suffer from this condition at an early stage of PD. These datasets are publicly accessible in the UCI Machine Learning Repository. The efficiency of the ISNDAM model was contrasted with that of seventy other models implemented in the WEKA workbench and was compared to the performance of previous studies. A statistical significance analysis was performed to verify that the performance differences between the compared models were statistically significant. The experimental findings allow us to affirm that the proposed improvement in the SNDAM algorithm, called ISNDAM, effectively increases the classification performance compared against well-known algorithms. ISNDAM achieves a classification accuracy of 99.48%, followed by ANN Levenberg–Marquardt with 95.89% and SVM RBF kernel with 88.21%, using Dataset 1. ISNDAM achieves a classification accuracy of 99.66%, followed by SVM IMF1 with 96.54% and RF IMF1 with 94.89%, using Dataset 2. The experimental findings show that ISNDAM achieves competitive performance on both datasets and that statistical significance tests confirm that ISNDAM delivers classification performance equivalent to that of models published in previous studies.

## 1. Introduction

Parkinson’s disease (PD) is a neurological condition that is chronic and worsens over time. It affects between 2 and 3 percent of the world’s population who are over the age of 65 [1,2], which presents a challenging diagnosis [3,4]. An accurate diagnosis is required to differentiate healthy individuals from PD patients. Studies have shown that PD can be diagnosed at early stages and that an early diagnosis of this neurodegenerative condition can lessen the impact of PD and improve patient’s living conditions [5,6]. At an early stage of this disease, a patient’s face may show no expression, and speech may become incomprehensible [7]. The first symptom may be a barely noticeable rhythmic shaking in only one hand. The symptoms of PD worsen as the condition progresses. Over the past few years, the use of computational intelligence (CI) techniques to diagnose PD has experienced rapid growth [8].

Almeida et al. [9] used feature extraction techniques on voice signals and machine learning methods for PD detection. They concluded that phonation analysis is a more efficient alternative to detect this progressive neurodegenerative disorder than speech tasks.

Polat and Nour [10] analyzed informative features from voice signals using a data-preprocessing method for PD recognition. They concluded that the proposed approach could be applied as a data-preprocessing method on computational intelligence algorithms for PD recognition, using informative features.

Pereira et al. [11] presented a methodical study of current enabling technologies that can be used to diagnose PD as well as to ameliorate the life quality of individuals with this condition. The authors also included a closer look into innovative and future approaches to achieve this goal.

Sakar et al. [12] presented a methodical study of voice signal processing for PD detection. The authors extracted features from voice signals of PD patients using a tunable wavelet transform approach (TQWT). The authors concluded that the performance of TQWT is superior to other cutting-edge speech signal processing approaches that are utilized in PD classification.

Pahuja and Nagabhushan [13] presented an analysis of the currently available supervised machine learning methods applied to Parkinson’s Disease recognition. They compared three types of supervised machine learning approaches: SVM, K-NN, and ANN. They concluded that a feed-forward ANN combined with the Levenberg–Marquardt method improves its performance and allows the highest classification rates to be achieved when applied to the voice dataset.

Alzubaidi et al. [14] investigated and provided a summary of neural networks applied to Parkinson’s disease diagnosis. The authors analyzed 91 studies to identify the function that a feed-forward artificial neural network plays in the process of PD diagnosis. The authors concluded that the early detection of PD through voice sample analysis occurs in a significant proportion of the 91 works analyzed. The authors also argue that ANNs are a viable option for performing this task.

Sechidis et al. [15] introduced a cross-domain transfer-learning model for speech emotion recognition and applied it to a Parkinsonian speech corpus. They evaluated distinctive voice patterns of patients with PD and concluded that there is a relationship between PD and emotional scores, where patients with this disease are often perceived as expressing an emotion of sadness.

Ma et al. [16] proposed an ensemble model for Parkinson speech recognition based on a dual-side learning ensemble that applies sample selection to remove useless samples and deep feature transformation to generate high-quality features for PD recognition. They concluded that the weighted fusion mechanism that merges classification algorithms is the crucial component that helps to achieve higher performance than the state-of-art relevant algorithms.

Haq et al. [17] presented a methodical review of the literature of deep learning methods frequently used in Parkinson detection. They explored current reviews with their advantages and limitations. They also presented numerical results comparison for current relevant methods.

Zhang et al. [18] introduced a feature called EDF-EMD and assessed its effectiveness on two datasets. The authors analyzed the frequency behavior of voice signals and concluded that the high-frequency segment of the speech signal provides relevant components related to the diagnosis and recognition of PD. They concluded that classification performance results are outstanding, using feature extraction from IMF1. The experiments suggest that the EDF-EMD feature can be utilized to recognize this medical condition effectively.

Mei et al. [19] presented a thorough analysis of the literature to study and enhance global understanding of supervised machine learning algorithms of practical application to Parkinson’s disorder recognition. The authors reviewed datasets, mathematical methods, data types, machine learning algorithms, data sources and the related results of 209 studies. The authors concluded that there is a strong potential of machine learning approaches in clinical decision making, which will lead to a more informed and methodical diagnosis of PD.

Ngo et al. [20] presented a methodical review of the literature of the last decade to investigate signal features and Parkinson’s disease recognition methods. They reported on the signal recording protocols, the different datasets used by supervised learning algorithms and signal analysis methods frequently used in Parkinson speech recognition. Based on classification and correlation results, they concluded that voice signal features as well as information extracted from speech recordings have a potential utility for Parkinson’s disorder recognition and assessment.

Quan et al. [21] presented a convolutional neural network (CNN) approach for Parkinson’s disorder recognition using voice samples. This approach is based on the successive application of CNN on voice samples. First, a 2D CNN is applied to obtain time series dynamic features, then a 1D CNN is used to obtain the dependencies between these time series. The authors concluded that higher frequencies of the Mel-spectrogram are less significant than low-frequency regions.

Madruga et al. [22] analyzed the impact of the variability that exists between different voice signal acquisition devices and the negative effects on voice signal recordings specifically oriented for Parkinson’s recognition. They proposed a methodology to increase the robustness against the variability between different devices. They concluded that this approach improves the capacity of supervised learning models to detect PD patients from healthy individuals, even using different voice signal acquisition devices.

Coelho et al. [23] analyzed the performance effects of Hjorth parameters extracted from electroencephalogram (EEG) signals for Parkinson’s disease detection. They evaluated the differences between healthy individuals’ and PD patients’ brain’s cerebral cortex. They concluded that there are substantial distinctions between healthy individuals’ and Parkinson’s disease patients’ brain’s lobes; thus, they can be used as Parkinson’s disease biomarkers.

Dixit et al. [24] presented a comprehensive overview of many AI-based machine learning and deep learning approaches to diagnose PD, as well as their impact on the development of new research areas. This review additionally provides an in-depth look at the current state of PD diagnostics as well as forthcoming applications of data-driven AI technology.

As evidenced by recent research, PD diagnosis continues to be a current topic of interest, and furthermore, its detection through voice samples using computational intelligence techniques is still a major challenge.

This paper presents an improvement to the smallest normalized difference associative memory (SNDAM) algorithm and shows performance measurement results achieved by this new proposal, called improved smallest normalized difference associative memory (ISNDAM), when it is applied to PD diagnosis. The experimental findings confirm that the proposed improvement in the SNDAM algorithm, called ISNDAM, is efficient and effectively increases the classification performance compared against well-known algorithms.

## 2. Materials and Methods

The learning matrix concept was conceived more than six decades ago [25,26,27], and since then, major research groups worldwide have shifted their focus to this promising research area [28,29,30,31,32]. The concept of an array that stores the relationship between patterns has evolved into what is known today as associative memories (AM).

Associative memories are used to simulate the basic learning activity of the human brain. Thus, this learning paradigm associates input and output patterns.

**Definition** **1.** *Let $x^{\mu }$ be the μ-th input pattern and $y^{\mu }$ be the μ-th output pattern, then the learning set is represented as follows:*

(1) $$\left\{\left(x^{1},y^{1}\right),\left(x^{2},y^{2}\right),...,\left(x^{\mu },y^{\mu }\right),...,\left(x^{p},y^{p}\right)\right\}$$

In this way, an associative memory M is created through the association of output patterns with input patterns. This stage is known as the training phase.

(2) xμ⟶M⟵yμ

Once associative memory M has been built, a test pattern $\tilde{x}$ will be presented to associative memory M to obtain an output pattern $y^{\vartheta }$ that will indicate the class label that corresponds to test pattern $\tilde{x}$. This stage is known as the operation phase.

(3) x˜⟶M⟶yϑ

### 2.1. Smallest Normalized Difference Associative Memory

This model was proposed in [33] to overcome the shortcomings of ABAM introduced in [34,35]. The ABAM model proposed two operators for its functionality: α and β. These operators were conceived in a binary space. Thus, there are certain drawbacks to this, such as the fact that all patterns have to be binary encoded [36], which increases the processing complexity but does not necessarily increase the recall capacity of the ABAM model [37].

Briefly stated, the main contribution of the SNDAM model is that it extended $\alpha_{R}$ and $\beta_{R}$ operators to the R domain, which reduced the complexity of the binary encoding of the fundamental set of patterns as well as the time consumed for model training, while the robustness to deal with subtractive, additive or combined alterations in input patterns was preserved.

**Definition** **2.** *The following describes the $\alpha_{R}$ operation:*

(4) $$\alpha_{R}\left(w,v\right)=w-v+1$$

**Definition** **3.** *The following describes the $\beta_{R}$ operator:*

(5) $$\beta_{R}\left(w,q\right)=\left\{\begin{array}{ccc} w & if & w\leq q\\ w+q-1 & if & w>q \end{array}\right.$$

The second contribution of this model is to use the smallest normalized difference to evaluate the similarity between an unknown recalled pattern and another one present in the training phase. This removes the ambiguity in the class label assignment; as a consequence, classification performance is improved.

The central core of SNDAM is based on operations between vectors to produce pattern associations, stored as a two-dimensional array (learning matrix) [33]. One crucial advantage of the SNDAM model is that it can fully recover the learning set when the learning phase is carried out in an autoassociative mode. Another aspect that distinguishes the SNDAM model is the robustness against additive or subtractive noise in training patterns. This turns out to be an advantage since an unknown pattern $x^{\omega }$ can be considered as a training pattern $x^{\mu }$ altered by noise (additive or subtractive).

**Definition** **4.** *Let M be the SNDAM MAX type, which is obtained as follows:*

(6) $$M=\overset{p}{\underset{\mu =1}{⋁}}\left(\alpha_{R}\left(x^{\mu },x^{\mu }\right)\right)$$

**Definition** **5.** *Let W be the SNDAM min type, which is obtained as follows:*

(7) $$W=\overset{p}{\underset{\mu =1}{⋀}}\left(\alpha_{R}\left(x^{\mu },x^{\mu }\right)\right)$$

**Definition** **6.** *Let A=R, let ⋁ be the maximum operator, and let $x^{MAX}\in A^{n}$ be an n-dimensional vector that contains the highest value of each of the n components of all the p instances in the learning set:*

(8) $$x_{i}^{MAX}=\overset{p}{\underset{\mu =1}{⋁}}x_{i}^{\mu }$$

**Definition** **7.** *Let $y^{\vartheta }$ be the recalled pattern from a given input instance $\tilde{x}$ that was not present in the learning set, using M:*

(9) $$y^{\vartheta }=⋀\left(\beta_{R}\left(M,\tilde{x}\right)\right)$$

**Definition** **8.** *Let $y^{\vartheta }$ be the recalled pattern from a given input instance $\tilde{x}$ that was not present in the learning set, using W:*

(10) $$y^{\vartheta }=⋁\left(\beta_{R}\left(W,\tilde{x}\right)\right)$$

**Definition** **9.** *Let $\delta^{\vartheta \mu }$ represent the magnitude of the similarity between each of the p instances $x^{\mu }$ in the learning set and the recalled pattern $y^{\vartheta }$:*

(11) $$\delta^{\vartheta \mu }=\sum_{i=1}^{n}\frac{\left|y_{i}^{\vartheta }-x_{i}^{\mu }\right|}{x_{i}^{max}}$$

### 2.2. Improved Smallest Normalized Difference Associative Memory

**Figure 1:**
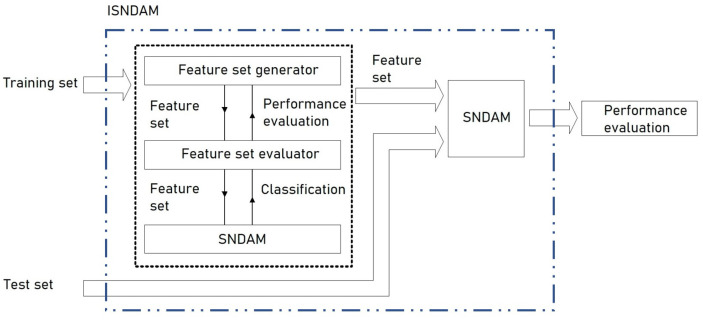
A wrapper-based feature selection approach applied to SNDAM. In a wrapper-based feature selection approach, performance is evaluated with all possible feature combinations. After an exhaustive search for relevant features, the set of features that increase classification performance is retained.

Even though the SNDAM model [33] overcame some of the shortcomings of ABAM [34,35], in this section, an improvement to the SNDAM model is proposed. This model is known as improved smallest normalized difference associative memory (ISNDAM), and it is based on the incorporation of a relevance identification phase, which is executed before the testing phase with unknown patterns, as is shown in Figure 1.

#### 2.2.1. ISNDAM Algorithm

**Figure 2:**
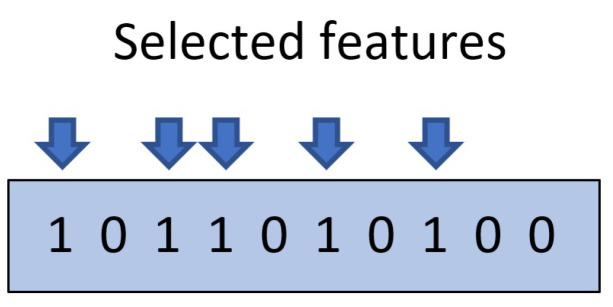
Example of a binary encoded relevance identification vector. The value 1 is assigned to features that contribute to improving classification performance, while the value 0 is assigned to those that do not.

The operation of ISNDAM requires three phases, namely: training, relevance identification and testing. As a result of the first phase, a learning matrix is obtained using those patterns present in the learning set. After that, an iterative search process is carried out in order to identify those characteristics that provide more information for the purposes of classification, as is shown in Figure 1. Those features that are identified as relevant are binary encoded and are stored in a reinforcement vector, as is shown in Figure 2. Finally, the smallest normalized difference is applied to test the unknown patterns; thus, the performance indicators of the proposed model are obtained.

#### 2.2.2. Training Phase

At first, a learning matrix M is built by associating all *p* instances in the learning set. After classifying these instances, a performance measure of the learning set is obtained. This first indicator sets the classification performance lower bound. Algorithm 1 describes the steps required to implement this phase.
**Algorithm 1:** Training Phase**Data: **$p,n,\{\left(x^{1},y^{1}\right),\left(x^{2},y^{2}\right),...,\left(x^{p},y^{p}\right)\}$**Result: **$M,y^{\vartheta }$Initialization:Step 1: Generate *p* matrices, one for each fundamental pattern association $\left(x^{\mu },x^{\mu }\right)$, using Equation (6).Step 2: Compare the previously generated *p* matrices, and keep the maximum value (⋁). The result is a learning matrix M that contains the maximum value of all *p* generated matrices.Step 3: Compare the *n* components of all the *p* instances in the learning set, and keep the maximum value (⋁), using Equation (8). The result is an *n*-dimensional vector that contains the highest value of each of the *n* components of all the the *p* instances $x^{\mu }$ in the learning set.Step 4: Recall all the *p* instances $x^{\mu }$ in the learning set, using Equation (9).Step 5: Obtain the magnitude of the similarity $\delta^{\vartheta \mu }$ between each of the *p* instances $x^{\mu }$ in the learning set and the recalled pattern $y^{\vartheta }$, using Equation (11).Step 6: Identify μ value, using the smallest normalized similarity value $\delta^{\vartheta \mu }$.Step 7: Assign $x^{\mu }$ class label to the recalled pattern $y^{\vartheta }$.End of Training Phase

#### 2.2.3. Relevance Identification Phase

The relevance identification phase consists of an iterative search process to identify and to select those features that improve ISNDAM classification performance. Algorithm 2 describes the steps required to implement this phase. The selected subset of features retains relevant information that sufficiently describes the problem to be effectively solved. This optimal subset of features is represented by a binary encoded *n*-dimensional vector. Those features that improve classifier performance are coded with a value of 1, while those features that negatively affect the performance of the classifier are coded with a value of 0, as is shown in Figure 2.

**Definition** **10.** *Let A={0,1} and let $e^{r}$ be a binary coded n-dimensional column vector that represents the identification of those relevant features that improve the performance of the model, with the r value represented in binary form:*

(12) $$e^{r}=\left(\begin{array}{c} e_{1}^{r}\\ e_{2}^{r}\\ \vdots \\ e_{n}^{r} \end{array}\right)\in A^{n}$$

**Algorithm 2:** Relevance Identification Phase

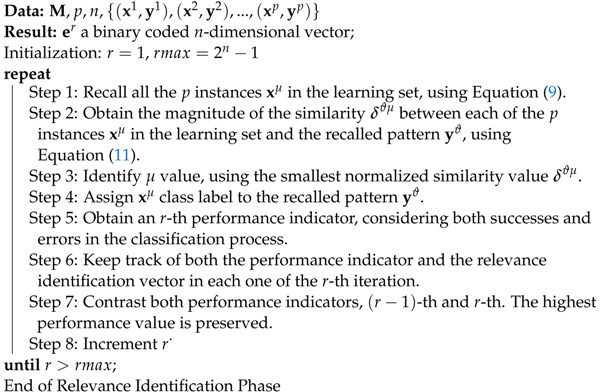



#### 2.2.4. Testing Phase

In this third phase, those features that benefit the performance of ISNDAM have already been identified. The relevance identification vector $e^{r}$ ignores those features that negatively affect the classification performance; thus, the testing phase is executed considering only relevant features. In this phase, test instances are recalled, and the smallest normalized difference value obtained is computed using all training patterns to assign a class label. Algorithm 3 describes the steps required to implement this phase.
**Algorithm 3:** Testing Phase**Data: **$M,p,n,e^{r},\tilde{x}$**Result: **$y^{\vartheta }$Initialization:Step 1: Use memory M, previously generated in the training phase, to recall $y^{\vartheta }$ from a given input instance $\tilde{x}$ that was not present in the learning set, using Equation (9).Step 2: Obtain the magnitude of the similarity $\delta^{\vartheta \mu }$ between each of the *p* instances $x^{\mu }$ in the learning set and the recalled pattern $y^{\vartheta }$, using Equation (11).Step 3: Identify μ value, using the smallest normalized similarity value $\delta^{\vartheta \mu }$.Step 4: Assign $x^{\mu }$ class label to the recalled pattern $y^{\vartheta }$.End of Testing Phase

#### 2.2.5. ISNDAM Numerical Example

In order to clarify the implementation of the ISNDAM model, the operation of the proposed model is illustrated by way of an example. Numeric values in our example correspond to patterns taken from the widely known iris plants dataset [38]. Each instance has four features, and there are an equal number of instances in each class, specifically three instances of each class.

**Example.**  *Given six instances in the learning set, each described by n = 4 features, where* $\left\{x^{1},x^{2},x^{3}\right\}$ *belong to class 1 and* $\left\{x^{4},x^{5},x^{6}\right\}$ *belong to class 2, recall all instances and obtain a classification performance measure.*

(13) $$\begin{array}{ccc} x^{1} & = & \left(\begin{array}{c} 5.1\\ 3.5\\ 1.4\\ 0.2 \end{array}\right)\;x^{2}=\left(\begin{array}{c} 4.9\\ 3.0\\ 1.4\\ 0.2 \end{array}\right)\;x^{3}=\left(\begin{array}{c} 4.7\\ 3.2\\ 1.3\\ 0.2 \end{array}\right) \end{array}$$

(14) $$\begin{array}{ccc} x^{4} & = & \left(\begin{array}{c} 7.0\\ 3.2\\ 4.7\\ 1.4 \end{array}\right)\;x^{5}=\left(\begin{array}{c} 6.4\\ 3.2\\ 4.5\\ 1.5 \end{array}\right)\;x^{6}=\left(\begin{array}{c} 6.9\\ 3.1\\ 4.9\\ 1.5 \end{array}\right) \end{array}$$

Generate *p* matrices, one for each fundamental pattern association $\left(x^{\mu },x^{\mu }\right)$, using Equation (6). Thus, each matrix element can be obtained using the following expression:

(15) $$m_{ij}=\alpha_{R}\left(x_{i}^{\mu },x_{j}^{\mu }\right)$$

Once all *p* matrices have been obtained, the maximum operator ⋁ is applied according to Equation (6). This means that each $m_{ij}$ component of each matrix must be compared and the maximum value is stored, so that finally a single matrix containing maximum values is obtained. In our example, the SNDAM autoassociative MAX-type obtained is as follows:

(16) $$M=\left(\begin{array}{cccc} 1 & 4.8 & 4.7 & 6.6\\ -0.5 & 1 & 3.1 & 4.3\\ -0.9 & 2.8 & 1 & 4.4\\ -3.5 & -0.6 & -0.1 & 1 \end{array}\right)$$

Use memory M, previously generated in the training phase, to recall $y^{\vartheta }$ from a given input instance $\tilde{x}$ that was not present in the learning set, using Equation (9). Then, obtain the magnitude of the similarity $\delta^{\vartheta \mu }$ between each of the *p* instances $x^{\mu }$ in the learning set and the recalled pattern $y^{\vartheta }$, using Equation (11). Thus, each component of the recalled pattern $y^{\vartheta }$ is obtained using the following expression:

(17) $$y_{i}^{\vartheta }=\overset{n}{\underset{j=1}{⋀}}\beta_{R}\left(m_{ij},{\tilde{x}}_{j}\right)$$

Firstly, the M matrix is taken, and the Beta operator $\beta_{R}$ is applied to the first instance to be tested. In our case, we take the first instance of the numerical example $x^{1}$

(18) $$y^{\vartheta }=\overset{n}{\underset{j=1}{⋀}}\left(\beta_{R}\left(\left(\begin{array}{cccc} 1.0 & 4.8 & 4.7 & 6.6\\ -0.5 & 1.0 & 3.1 & 4.3\\ -0.9 & 2.8 & 1.0 & 4.4\\ -3.5 & -0.6 & -0.1 & 1.0 \end{array}\right)\;\left(\begin{array}{c} 5.1\\ 3.5\\ 1.4\\ 0.2 \end{array}\right)\right)\right)$$

Subsequently, the minimum operator ⋀ is applied, and as a result, we obtain vector $y^{\vartheta }$.

(19) $$y^{\vartheta }=\left(\begin{array}{ccccccc} 5.1 & \wedge & 7.3 & \wedge & 5.1 & \wedge & 5.8\\ 3.6 & \wedge & 3.5 & \wedge & 3.5 & \wedge & 3.5\\ 3.2 & \wedge & 5.3 & \wedge & 1.4 & \wedge & 3.6\\ 0.6 & \wedge & 1.9 & \wedge & 0.3 & \wedge & 0.2 \end{array}\right)=\left(\begin{array}{c} 5.1\\ 3.5\\ 1.4\\ 0.2 \end{array}\right)\to y^{\vartheta }=\left(\begin{array}{c} 5.1\\ 3.5\\ 1.4\\ 0.2 \end{array}\right)$$

Once vector $y^{\vartheta }$ has been recalled, what follows is to obtain the magnitude of the similarity $\delta^{\vartheta \mu }$ between each of the *p* instances $x^{\mu }$ in the learning set and the recalled pattern $y^{\vartheta }$, using Equation (11)

For this example, $y^{\vartheta }$ will be compared with $\left\{x^{1},x^{2},\ldots ,x^{6}\right\}$  and the smallest normalized difference is obtained, according to (11).

(20) δ118.41E-18δ120.30δ130.48δ142.72δ152.09δ162.65

As can be seen, the smallest normalized difference $\delta^{\vartheta \mu }$ between $y^{\vartheta }$ and each of the patterns present in the training phase occurs in the first position $\delta^{11}$. This result is intuitive because the recalled values of $y^{\vartheta }$ are present in the $x^{1}$ pattern. Therefore, since $y^{\vartheta }$ has a greater similarity with $x^{1}$, the class label of $x^{1}$ is applied to $y^{\vartheta }$. This implies that $y^{\vartheta }$ belongs to class 1. It can be verified that all patterns $\left\{x^{1},x^{2},\ldots ,x^{6}\right\}$ that are present in the training phase are correctly recalled, that is, recalled patterns $\left\{y^{1},y^{2},y^{3}\right\}$ belong to class 1 and $\left\{y^{4},y^{5},y^{6}\right\}$ belong to class 2.

What follows is to test the ISNDAM model with unknown patterns. At this stage, we are interested in obtaining the class label that corresponds to test pattern $\tilde{x}$, which is achieved by measuring the similarity between each one of those patterns that were present in the training phase and the recalled pattern ${\tilde{y}}^{\vartheta }$.

The test pattern $\tilde{x}$ is the following:

(21) $$\tilde{x}=\left(\begin{array}{c} 6.8\\ 3.2\\ 4.7\\ 1.5 \end{array}\right)$$

Firstly, the M matrix is taken, and the Beta operator $\beta_{R}$ is applied to the unknown pattern $\tilde{x}$ to be tested.

(22) $${\tilde{y}}^{\vartheta }=\overset{n}{\underset{j=1}{⋀}}\left(\beta_{R}\left(\left(\begin{array}{cccc} 1 & 4.8 & 4.7 & 6.6\\ -0.5 & 1 & 3.1 & 4.3\\ -0.9 & 2.8 & 1 & 4.4\\ -3.5 & -0.6 & -0.1 & 1 \end{array}\right)\;\left(\begin{array}{c} 6.8\\ 3.2\\ 4.7\\ 1.5 \end{array}\right)\right)\right)$$

Subsequently, the minimum operator ⋀ is applied, and as a result, we obtain vector ${\tilde{y}}^{\vartheta }$.

(23) $${\tilde{y}}^{\vartheta }=\left(\begin{array}{ccccccc} 6.8 & \wedge & 7.0 & \wedge & 8.4 & \wedge & 7.1\\ 5.3 & \wedge & 3.2 & \wedge & 6.8 & \wedge & 4.8\\ 4.9 & \wedge & 5.0 & \wedge & 4.7 & \wedge & 4.9\\ 2.3 & \wedge & 1.6 & \wedge & 3.6 & \wedge & 1.5 \end{array}\right)=\left(\begin{array}{c} 6.8\\ 3.2\\ 4.7\\ 1.5 \end{array}\right)\to {\tilde{y}}^{\vartheta }=\left(\begin{array}{c} 6.8\\ 3.2\\ 4.7\\ 1.5 \end{array}\right)$$

Once vector ${\tilde{y}}^{\vartheta }$ has been recalled, what follows is to obtain the smallest normalized difference $\delta^{\vartheta \mu }$ between each pattern in the learning set and the recalled pattern ${\tilde{y}}^{\vartheta }$. For these purposes, ${\tilde{y}}^{\vartheta }$ will be compared with $\left\{x^{1},x^{2},\ldots ,x^{6}\right\}$, and the smallest normalized difference will be obtained, according to Equation (11).

(24) δ112.49δ122.79δ132.97δ140.23δ150.39δ160.16

As can be seen, the smallest normalized difference $\delta^{\vartheta \mu }$ between ${\tilde{y}}^{\vartheta }$ and each of the patterns present in the training phase occurs in the sixth position $\delta^{16}$. Therefore, since ${\tilde{y}}^{\vartheta }$ has a greater similarity with $x^{6}$, the class label of $x^{6}$ is applied to ${\tilde{y}}^{\vartheta }$. This implies that ${\tilde{y}}^{\vartheta }$ belongs to class 2.

In summary, the ISNDAM model takes advantage of the properties of the learning matrices trained in auto-associative mode and uses the smallest normalized difference $\delta^{\vartheta \mu }$ to measure the similarity between those patterns present in the training phase and some unknown pattern ${\tilde{y}}^{\vartheta }$.

- One of the contributions of the ISNDAM model is the simplification of the Beta operator $\beta_{R}$ to a single case, which eliminates ambiguity in class assignment and consequently increases classification performance.
- The second contribution of this model is the relevance identification phase, which identifies all the relevant characteristics for classification purposes through a wrapper-based feature selection approach applied to SNDAM, as is shown in Figure 1.

### 2.3. Datasets

In this section, information about the datasets used for the validation of the performance measurements of ISNDAM model is provided. Table 1 summarizes the most relevant characteristics of them. More information about these datasets can be found in public data repositories [39,40].

- The first dataset was created at Oxford University to differentiate PD patients from healthy individuals. It was generated from voice signal analysis measurements from thirty-one individuals, where twenty-three have PD. This dataset consists of 195 instances with 23 attributes. More details on how the recordings were gathered as well as the feature extraction process can be found at [41].
- The second dataset was created at Istanbul University, from voice recordings of forty individuals, where twenty of them are healthy individuals and the remaining twenty patients have PD. This dataset consists of 1040 instances with 27 attributes. More details on how the recordings were gathered as well as the feature extraction process can be found at [12].

### 2.4. Performance Metrics

In this section, a succinct description of each performance metric is presented. First, let *P* be the number of individuals who have a certain condition, let *N* be the number of individuals who do not have such condition, let TP be the true positive value that refers to a test outcome that accurately indicates the existence of a particular condition in a patient, let FP be the false positive value that refers to a test outcome that wrongly indicates the existence of a particular condition in a patient, let TN be the true negative value that refers to a test outcome that accurately indicates the non-existence of a certain condition in a patient, and let FN be the false negative value that refers to a test outcome that wrongly indicates the non-existence of a certain condition in a patient.

A confusion matrix contrasts predicted and actual classes to visually show the performance of a model [42]. A particular case of confusion matrices appears when there is a problem where there are only two classes (negative and positive). This is exemplified as follows: (25)
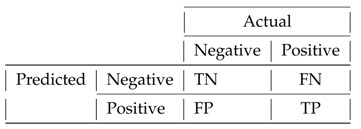

- Sensitivity represents a test’s capacity to accurately identify all individuals who have a certain condition, also known as recall:
  (26) $$Sensitivity=TPR=\frac{TP}{TP+FN}$$
- Accuracy refers to a model’s performance. It is computed as the proportion of tests that were properly predicted by all of the predictions:
  (27) $$Accuracy=\frac{TN+TP}{N+P}$$
  (28) $$Accuracy=\frac{TN+TP}{TP+FP+FN+TN}$$
- Specificity indicates a test’s capacity to correctly detect every individual who does not have a certain condition:
  (29) $$Specificity=\frac{TN}{FP+TN}$$
- False positive rate (FPR) indicates a test’s capacity to incorrectly detect healthy individuals who do not have a certain condition:
  (30) $$FPR=\frac{FP}{FP+TN}=1-TNR$$
- Precision refers to the reliability of a model in predicting a positive test result. It represents the proportion of tests that were accurately predicted as positive to the total number of tests that were forecast as positive:
  (31) $$Precision=\frac{TP}{FP+TP}$$
- Area under the ROC curve (AUC) represents how well a binary classification algorithm is able to identify the difference between two classes [43]:
  (32) $$AUC=\frac{1+TPR-FPR}{2}=\frac{TPR+TNR}{2}$$
  (33) $$AUC=\frac{TPR+TNR}{2}=\frac{Sensitivity+Specificity}{2}$$
- Geometric mean (G-Mean) estimates the balance of classification performance between the minority and majority classes:
  (34) $$G-Mean=\sqrt{Specificity\times Sensitivity}$$
- F1-score is determined by finding the harmonic mean of the assessments for sensitivity and precision. It represents sensitivity and precision into a single statistic in a symmetrical form:
  (35) $$F1-Score=2\times \left(\frac{Sensitivity\times Precision}{Sensitivity+Precision}\right)$$

### 2.5. Statistical Hypothesis Tests

In statistical hypothesis testing, the objective is to provide evidence that the performance metric findings are representative of the overall behavior of classifiers. In addition to performance assessments obtained from the metrics described in Section 2.4, there are two additional aspects to consider: the first is whether the reported findings can be attributed to the properties of the classifiers or whether they could have occurred by chance, and the second is whether the apparent superiority in performance of one algorithm over another is not due to random selection of training or testing samples. To address these two factors, a statistical significance analysis is required [33].

As suggested in [44,45], the two-matched-samples t test can be useful to find whether the difference between two means is meaningful [46], which is applicable in the case of two independent samples [47]. Hypothesis testing consists of assuming two hypotheses: an alternative hypothesis, $H_{1}$, and a null hypothesis, $H_{0}$, which is generally the opposite of what we want to prove. Rejecting the null hypothesis implies, based on evidence, that the alternative hypothesis is accepted, which gives us confidence in believing that the observations were not by chance. Algorithm 4 outlines the steps necessary to conduct a statistical significance test.

**Definition** **11.** *Let $H_{0}$ be a null hypothesis; it is assumed to be true until evidence indicates otherwise and particularly states: classifiers A and B have the same performance.*

**Algorithm 4:** Statistical Significance Test

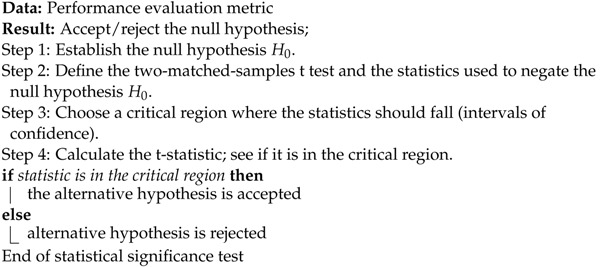



## 3. Results

The performance metrics, briefly described in Section 2.4, were computed for each compared algorithm using two datasets: Dataset 1 [41] and Dataset 2 [12]. The comparison of the obtained results is divided into two sections.

- First: The efficiency of ISNDAM was evaluated and compared to the efficiency of seventy different models that are included in the WEKA workbench [48,49]. All experiments were performed using 5 × 2 cross-validation, as suggested in [50,51]. Additionally, a statistical significance test was conducted to verify if there existed statistically significant differences in the performance of each compared algorithm included in the WEKA workbench. Performance outcomes using Dataset 1 are shown in Table 2, while Table 3 shows the results of the statistical significance analysis for Dataset 1. Similarly, performance outcomes using Dataset 2 are shown in Table 4, while the statistical significance analysis for Dataset 2 is presented in Table 5.
- Second: The performance of ISNDAM was compared to that of previous studies [13,19,24,52] using Dataset 1, as well as to that of previous studies [11,12,18] using Dataset 2. The performance results of ISNDAM compared to that of previous studies [13,24] using Dataset 1 are shown in Table 6. In the same way, the performance results of ISNDAM compared to that of previous studies [19,52] using Dataset 1 are shown in Table 7. Similarly, the performance results of ISNDAM compared to that of previous studies [11,12,18] using Dataset 2 are shown in Table 8.

As is shown in Table 2 and Table 4, several performance metrics, briefly described in Section 2.4, were used for comparison purposes: accuracy, G-Mean, specificity, F1-score, precision, AUC and sensitivity. Statistical significance test results for the paired measurements between the ISNDAM classification accuracy results and the performance outcomes of all of the algorithms compared are presented in Table 3 and Table 5. In accordance with Table 2, it is clear that ISNDAM achieves the best performance, followed by SNDAM and IBk, using Dataset 1. The first two models belong to the associative model-based classifier family, while IBk belongs to the lazy classifiers family. The default number of nearest neighbors used by IBk is (k = 1), while the default distance function is the Euclidean. As suggested in [44], the two-matched-samples t test can be useful in finding if the difference between two means is meaningful [46]. Table 3 shows the statistical significance test results using Dataset 1 for the paired measurements between the ISNDAM classification accuracy and the classification assessment results of each compared algorithm, with a confidence interval percentage of 95%, where *p* < 0.05 establishes the statistical significance threshold. Given the conditions of the two-matched-samples t test, classification accuracy results pairwise comparisons have to be made [45]. Therefore, we have seven *p* value measurements to be evaluated. Thus, a *p* value smaller than the statistical significance threshold implies that less evidence exists to support the null hypothesis. In each pairwise comparison of Table 3, it can be observed that the alternative hypothesis is accepted. This allows us to affirm that there exists a statistically significant difference between ISNDAM classification accuracy and the performance achieved by all the other compared models. This implies that even when performance differences are small, they are not due to random selection of training or testing samples; that is, the performance of the ISNDAM model is superior to the performance achieved by the other models that appear in Table 2 using Dataset 1 and 5 × 2 cross-validation.

Following the same analysis scheme, according to Table 4, it is clear that ISNDAM achieves the best performance, followed by SNDAM and random forest, using Dataset 2. The first two models belong to the associative model-based classifier family, while random forest belongs to the decision tree classifiers family. Table 5 shows the statistical significance test results using Dataset 2 for the paired measurements between the ISNDAM classification accuracy and the classification assessment results of each compared algorithm, with a confidence interval percentage of 95%, where *p* < 0.05 establishes the statistical significance threshold. We have seven *p* value measurements to be evaluated. In each pairwise comparison of Table 5, it can be observed that the alternative hypothesis is accepted. This allows us to affirm that there exists a statistically significant difference between ISNDAM classification accuracy and the performance achieved by all the other compared models. This implies that the performance of the ISNDAM model is superior to the performance achieved by the other models that appear in Table 4 using Dataset 2 and 5 × 2 cross-validation.

With the purpose of expanding the experimental outcomes interpretation, the performance of ISNDAM was compared to that of previous studies [13,19,24,52] using Dataset 1, as well as to that of previous studies [11,12,18] using Dataset 2.

As it is shown in Table 6 the performance of ISNDAM was compared to that of previous studies [13,24]. It is clear that ISNDAM achieves the best performance with a classification accuracy of 99.48%, followed by ANN Levenberg–Marquardt with 95.89% and SVM RBF kernel with 88.21%, using Dataset 1.

Similarly, Table 7 shows the performance of ISNDAM compared to that of previous studies [19,52]. It is clear that ISNDAM achieves the best performance with a classification accuracy of 99.48% using the raw features of Dataset 1. However, if the weighted features approach [52] is applied to Dataset 1, the highest classification accuracy is achieved by LS-SVM, PNN and GRNN.

In the same way, Table 8 shows the classification accuracy of ISNDAM compared to that of previous studies [11,12,18]. It is clear that ISNDAM achieves the best performance with a classification accuracy of 99.66%, followed by SVM IMF1 with 96.54% and RF IMF1 with 94.89%, using Dataset 2.

## 4. Discussion

One of the primary goals that this study intends to accomplish focuses on the development of a machine learning model that assists the Parkinson’s disease detection process with competitive performance. To achieve this, it was necessary to show, through frequently used performance metrics, that a slight modification to the SNDAM model can improve PD detection from voice samples.

Another objective was to find whether the performance of the ISNDAM model differed significantly from that of other machine learning models. The fulfillment of this objective was accomplished using two computational platforms, namely: the WEKA workbench and IBM SPSS software platform. It was possible to verify that the proposed model presents a superior classification performance when compared to other machine learning models implemented in the WEKA workbench. This statement is based on a statistical significance analysis, where in all pairwise comparisons, the null hypothesis is rejected.

Another objective was to compare the performance of the ISNDAM model with classification models published in previous studies. For such purposes and in order to conduct a coherent comparison, the performance analysis was restricted to classification algorithms that used the same datasets and cross-validation schemes; therefore, classification performance of ISNDAM was compared to that of previous studies [13,19,24,52] using Dataset 1, as well as to that of previous studies [11,12,18] using Dataset 2.

While using Dataset 1, the ISNDAM model performance was superior in the following studies: Pahuja and Nagabhushan [13] and Dixit et al. [24].

Using raw features of Dataset 1, the performance of the ISNDAM model was superior in all the cases that appear in the following studies: Hariharan et al. [52] and Mei et al. [19]. However, using weighted features of Dataset 1, the highest classification accuracy was achieved by LS-SVM, PNN and GRNN.

While using Dataset 2, the ISNDAM model performance was superior in each of the following studies: Pereira et al. [11], Sakar et al. [12] and Zhang et al. [18].

There are several reasons why the proposed model offers competitive experimental performance. The first is because of its great learning capacity, specifically because it has been trained in autoassociative mode. The second is because of its high tolerance for noise in test patterns. The third is the ability of the proposed model to retrieve patterns when the associative memory has been trained in autoassociative mode. The last and perhaps the most important is the use of the smallest normalized distance, which helps to retrieve the class label of a pattern that was present in the training phase and assign it to an unknown (test) pattern that has the highest similarity with respect to some instance that was present in the training phase. It basically assigns a class label considering the maximum similarity between the unknown instance and the training instances.

## 5. Conclusions

This paper introduces an efficient method for performing Parkinson’s disease detection. This model is known as improved smallest normalized difference associative memory (ISNDAM) algorithm. It is the outcome of an improvement to the original SNDAM model. A feature selection stage was added to the original SNDAM model, which enhances the classification performance.

After performing an analysis on the outcomes of the experimental phase, it can be affirmed that the ISNDAM algorithm is an efficient and effective option for detecting Parkinson’s disease from voice samples.

## Figures and Tables

**Table 1:** Main attributes of the datasets used for performance measurement validation.

No.	Dataset	Instances	Attributes
1.	Parkinson’s Dataset (University of Oxford) [41]	195	23
2.	Parkinson’s Disease Classification Dataset (Istanbul University) [12]	1040	27

**Table 2:** Parkinson’s disease classification using Dataset 1, 5 × 2 cross-validation. The highest performance metric value is highlighted in boldface.

No.	Algorithm	Dataset 1					
		Accuracy	Sensitivity	Specificity	Precision	AUC	G-Mean
1.	IBk	96.41	96.60	95.83	98.61	96.22	96.21
2.	NaiveBayes	69.23	61.90	91.67	95.79	76.79	75.32
3.	MultilayerPerceptron	90.77	92.52	85.42	95.10	88.97	88.89
4.	RandomForest	91.79	95.92	79.17	93.38	87.54	87.14
5.	RandomTree	84.62	89.80	68.75	89.80	79.27	78.57
6.	SMO	87.18	99.32	50.00	85.88	74.66	70.46
7.	SNDAM	96.61	96.93	96.83	98.63	96.88	96.87
★	ISNDAM	**99.48**	**99.98**	**99.31**	**98.75**	**99.65**	**99.64**

**Table 3:** Statistical hypothesis tests using Dataset 1: Two-matched-samples *t* test, confidence interval percentage of 95%, *p* < 0.05 establishes statistical significance threshold.

No.	Compared Algorithms	Dataset 1		
		*p* Value	Null Hypothesis	Alternative Hypothesis
1.	ISNDAM—IBk	4.82 × 10${}^{-14}$	✗	✓
2.	ISNDAM—NaiveBayes	2.20 × 10${}^{-16}$	✗	✓
3.	ISNDAM—MultilayerPerceptron	5.10 × 10${}^{-12}$	✗	✓
4.	ISNDAM—RandomForest	4.41 × 10${}^{-13}$	✗	✓
5.	ISNDAM—RandomTree	7.51 × 10${}^{-14}$	✗	✓
6.	ISNDAM—SMO	9.31 × 10${}^{-11}$	✗	✓
7.	ISNDAM—SNDAM	1.37 × 10${}^{-04}$	✗	✓

**Table 4:** Parkinson’s disease classification using Dataset 2, 5 × 2 cross-validation. The highest performance metric value is highlighted in boldface.

No.	Algorithm	Dataset 2					
		Accuracy	Sensitivity	Specificity	Precision	AUC	G-Mean
1.	IBk	67.96	72.24	62.31	71.72	67.27	67.09
2.	NaiveBayes	59.85	73.98	41.15	62.45	57.57	55.17
3.	MultilayerPerceptron	68.05	69.91	65.58	72.88	67.74	67.71
4.	RandomForest	73.10	79.94	64.04	74.63	71.99	71.54
5.	RandomTree	66.14	71.80	58.65	69.68	65.23	64.89
6.	SMO	66.23	84.74	41.73	65.80	63.23	59.46
7.	SNDAM	98.28	98.18	98.19	97.95	98.19	98.19
★	ISNDAM	**99.66**	**99.23**	**99.98**	**99.98**	**99.61**	**99.60**

**Table 5:** Statistical hypothesis tests using Dataset 2: Two-matched-samples *t* test, confidence interval percentage of 95%, *p* < 0.05 establishes statistical significance threshold.

No.	Compared Algorithms	Dataset 2		
		*p* Value	Null Hypothesis	Alternative Hypothesis
1.	ISNDAM—IBk	1.73 × 10${}^{-14}$	✗	✓
2.	ISNDAM—NaiveBayes	2.20 × 10${}^{-16}$	✗	✓
3.	ISNDAM—MultilayerPerceptron	5.86 × 10${}^{-13}$	✗	✓
4.	ISNDAM—RandomForest	1.12 × 10${}^{-11}$	✗	✓
5.	ISNDAM—RandomTree	1.08 × 10${}^{-12}$	✗	✓
6.	ISNDAM—SMO	6.54 × 10${}^{-13}$	✗	✓
7.	ISNDAM—SNDAM	1.95 × 10${}^{-03}$	✗	✓

**Table 6:** Comparing the effectiveness of our proposed method to previous studies [13,24] using Dataset 1. The highest performance metric value is highlighted in boldface.

No.	Algorithms	Dataset 1				
		Accuracy	Sensitivity	Specificity	AUC	G-Mean
1.	KNN Cityblock distance	69.74	66.67	70.75	68.71	68.68
2.	KNN Euclidean distance	72.31	68.75	73.47	71.11	71.07
3.	SVM Polynomial kernel	81.03	79.17	87.76	83.46	83.35
4.	SVM Linear kernel	82.90	87.33	78.56	82.94	82.83
5.	SVM RBF kernel	88.21	91.67	77.55	84.61	84.31
6.	ANN Scaled conjugate gradient	85.12	70.00	96.59	83.30	82.23
7.	ANN Levenberg–Marquardt	95.89	93.75	96.59	95.17	95.16
★	ISNDAM	**99.48**	**99.98**	**99.31**	**99.65**	**99.64**

**Table 7:** Comparing the effectiveness of our proposed method to previous studies [19,52] using Dataset 1. Raw features (RF), weighted features (WF), general regression neural network (GRNN), least-square support vector machine (LS-SVM) and probabilistic neural network (PNN). The highest performance metric value is highlighted in boldface.

No.	Algorithms	Dataset 1					
		Feature Reduction/Selection	Accuracy	Sensitivity	Specificity	AUC	G-Mean
1.	LS-SVM	22 RF	95.38	96.44	92.05	94.24	94.21
2.	LS-SVM	22 WF	**100.00**	**100.00**	**100.00**	**100.00**	**100.00**
3.	PNN	22 RF	95.49	97.99	88.47	93.23	93.10
4.	PNN	22 WF	**100.00**	**100.00**	**100.00**	**100.00**	**100.00**
5.	GRNN	22 RF	95.49	98.13	88.20	93.16	93.03
6.	GRNN	22 WF	**100.00**	**100.00**	**100.00**	**100.00**	**100.00**
★	ISNDAM	22 RF	99.48	99.98	99.31	99.65	99.64

**Table 8:** Comparing the effectiveness of our proposed method to previous studies [11,12,18] using Dataset 2. Random forests (RF), support vector machine (SVM), intrinsic mode functions (IMF). The highest performance metric value is highlighted in boldface.

No.	Algorithms	Dataset 2			
		Accuracy	Sensitivity	Precision	F1
1.	SVM IMF1	96.54	92.01	99.45	95.58
2.	SVM IMF2	93.16	87.76	95.71	91.56
3.	SVM IMF3	84.68	82.23	83.24	82.73
4.	SVM IMF4	83.00	75.00	81.49	78.11
5.	SVM IMF5	77.54	74.89	72.89	73.87
6.	SVM IMF6	63.33	54.25	63.05	58.31
7.	RF IMF1	94.89	94.78	95.23	95.00
8.	RF IMF2	91.67	94.12	84.21	88.88
9.	RF IMF3	81.52	70.00	88.44	78.14
10.	RF IMF4	79.67	66.00	81.25	72.83
11.	RF IMF5	77.08	70.22	73.68	71.90
12.	RF IMF6	72.92	60.00	70.59	64.86
★	ISNDAM	**99.66**	**99.23**	**99.98**	**99.60**

## Data Availability

Not applicable.
